# Application of a Perception Neuron^®^ System in Simulation-Based Surgical Training

**DOI:** 10.3390/jcm8010124

**Published:** 2019-01-21

**Authors:** Hyun Soo Kim, Nhayoung Hong, Myungjoon Kim, Sang Gab Yoon, Hyeong Won Yu, Hyoun-Joong Kong, Su-Jin Kim, Young Jun Chai, Hyung Jin Choi, June Young Choi, Kyu Eun Lee, Sungwan Kim, Hee Chan Kim

**Affiliations:** 1 Department of Surgery, Seoul National University Hospital, 101 Daehak-ro, Jongno-gu, Seoul 03080, Korea; surgeonhyunsoo83@gmail.com (H.S.K.); sgyoon8282@gmail.com (S.G.Y.); 2 Interdisciplinary Program in Bioengineering, Seoul National University, 101 Daehak-ro, Jongno-gu, Seoul 03080, Korea; jhong407@naver.com; 3 Korea Electrotechnology Research Institute, 111, Hanggaul-ro, Sangnok-gu, Ansan-si 15588, Korea; kimmj08@snu.ac.kr; 4 Department of Surgery, Seoul National University Bundang Hospital, 82, Gumi-ro 173 Beon-gil, Bundang-gu, Seongnam-si, Gyeonggi-do 13620, Korea; juneychoi@snubh.org; 5 Department of Biomedical Engineering, Chungnam National University College of Medicine, 266 Munhwa-ro, Jung-gu, Daejeon 36015, Korea; gongcop@cnu.ac.kr; 6 Department of Surgery, Seoul National University Hospital and College of Medicine, 101 Daehak-ro, Jongno-gu, Seoul 03080, Korea; su.jin.kim.md@gmail.com (S.-J.K.); kyueunlee@snu.ac.kr (K.E.L.); 7 Department of Surgery, Seoul National University Boramae Medical Center, 20 Boramae-ro 5-gil, Dongjak-gu, Seoul 07061, Korea; kevinjoon@naver.com; 8 Department of Anatomy and Cell Biology, Neuroscience Research Institute, Wide River Institute of Immunology, BK21Plus Biomedical Science Project, Seoul National University College of Medicine, 101 Daehak-ro, Jongno-gu, Seoul 03080, Korea; hjchoi@snu.ac.kr; 9 Department of Biomedical Engineering, Seoul National University College of Medicine, 101 Daehak-ro, Jongno-gu, Seoul 03080, Korea; sungwan@snu.ac.kr (S.K.); hckim@snu.ac.kr (H.C.K.)

**Keywords:** Surgical training, simulation, motion capture, thyroidectomy training model, perception neuron

## Abstract

While multiple studies show that simulation methods help in educating surgical trainees, few studies have focused on developing systems that help trainees to adopt the most effective body motions. This is the first study to use a Perception Neuron^®^ system to evaluate the relationship between body motions and simulation scores. Ten medical students participated in this study. All completed two standard tasks with da Vinci Skills Simulator (dVSS) and five standard tasks with thyroidectomy training model. This was repeated. Thyroidectomy training was conducted while participants wore a perception neuron. Motion capture (MC) score that indicated how long the tasks took to complete and each participant’s economy-of-motion that was used was calculated. Correlations between the three scores were assessed by Pearson’s correlation analyses. The 20 trials were categorized as low, moderate, and high overall-proficiency by summing the training model, dVSS, and MC scores. The difference between the low and high overall-proficiency trials in terms of economy-of-motion of the left or right hand was assessed by two-tailed *t*-test. Relative to cycle 1, the training model, dVSS, and MC scores all increased significantly in cycle 2. Three scores correlated significantly with each other. Six, eight, and six trials were classified as low, moderate, and high overall-proficiency, respectively. Low- and high-scoring trials differed significantly in terms of right (dominant) hand economy-of-motion (675.2 mm and 369.4 mm, respectively) (*p* = 0.043). Perception Neuron^®^ system can be applied to simulation-based training of surgical trainees. The motion analysis score is related to the traditional scoring system.

## 1. Introduction

Traditional medical training, including surgical training, is based on the apprenticeship method, where trainees are directly or indirectly taught the requisite skills and knowledge by masters in the field [1,2]. However, in recent years, pressure to decrease the work hours of residents has significantly reduced the time devoted to their hands-on education [3]. This reduction of training opportunities could increase medical malpractice claims, which are already a significant problem in many countries. For example, 7.4% of physicians in the United States of America (USA) had malpractice claims made against them in 1990–2011, and 1.6% paid claim costs during this period [4]. Residents, like specialists, are also liable for medical claims; Thiels et al. showed that in the USA in 2005–2015, there were 87 medical malpractice lawsuits that involved surgical residents [5]. These findings have led to increased focus on the appropriate education of surgical residents.

To address this issue and provide a good quality of surgical educations, many departments, including surgery, obstetrics, and urology, have introduced simulation-based surgical education methods [6,7,8]. Several studies show that adding simulation-based surgical education to the curriculum significantly increases the surgical skills of trainees [9,10,11].

To date, various educational aspects of surgical simulators, training boxes, and training mannequins have been researched extensively, including how well they recreate the surgical setting and how effectively they improve the surgical skills of the trainees [12,13]. Much less attention has been devoted to the development of systems that help trainees to correct their body motions during simulation. Only two studies to date have examined how the body movements of experienced operators using a box trainer or robotic simulator differ from those of trainees; for this, a motion capture system was used, and the movements were analyzed [14,15].

Bilateral axillo-breast approach (BABA) robotic thyroidectomy is an operation that requires a very high level of training [16]. Recently, Yu et al. reported that surgical training using the BABA training model is as effective as the conventional da Vinci Skills Simulator (dVSS) in terms of teaching the requisite skills for BABA robotic thyroidectomy [17]. However, studies on the body movements during use of the BABA training model and the dVSS, and how they correlate with the simulation scores, have not been performed. Such studies would be valuable because they would show which body movements best improve the simulation scores.

In the present study, we used Perception Neuron^®^, a recently introduced motion capture system, to analyze the body movements of participants who trained with the BABA training model. The BABA surgical skill and body motion capture scores which was developed in this study were compared with the dVSS scores. In addition, the training trials that exhibited high and low overall proficiency were compared in terms of body movements in three dimensions (3D) using *t*-test.

## 2. Materials and Methods

### 2.1. Participants and Study Design

**Figure 1:**
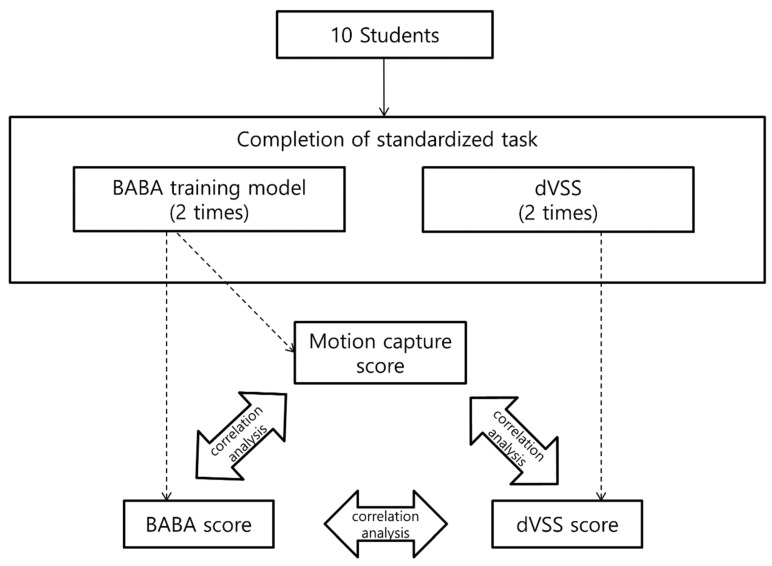
Study design. BABA: bilateral axillo-breast approach; dVSS: da Vinci Skills Simulator.

This prospective cross-sectional study was conducted at Seoul National University Hospital in 2017. The participants were recruited by a public meeting for medical students, during which the study was described. Medical students who volunteered to participate in the research were enrolled in the study after signing an agreement. The students trained on the dVSS once and then underwent training with the BABA training model. The dVSS score for two standard tasks and the BABA score were recorded. The BABA score indicated how well the trainee performed five standard BABA surgery tasks. It has been reported previously [17] and is described in detail below. The motions of the participants while they were training with the BABA training model were captured with Perception Neuron^®^, and the motion capture (MC) score was calculated. This score was invented in this study and is described in detail below. Thereafter, training with the dVSS and then the BABA training model was performed a second time. The dVSS, BABA, and MC scores were calculated again (Figure 1). The study protocol was approved by the Institutional Review Board of Seoul National University Hospital (H-1703-124-840). Ten students voluntarily gave their written consent to participate in the study. Before participating, all students identified the hand they use for handwriting and eating. In all cases, the dominant hand was the right hand.

### 2.2. BABA Training Model and Standardized Tasks

**Figure 2:**
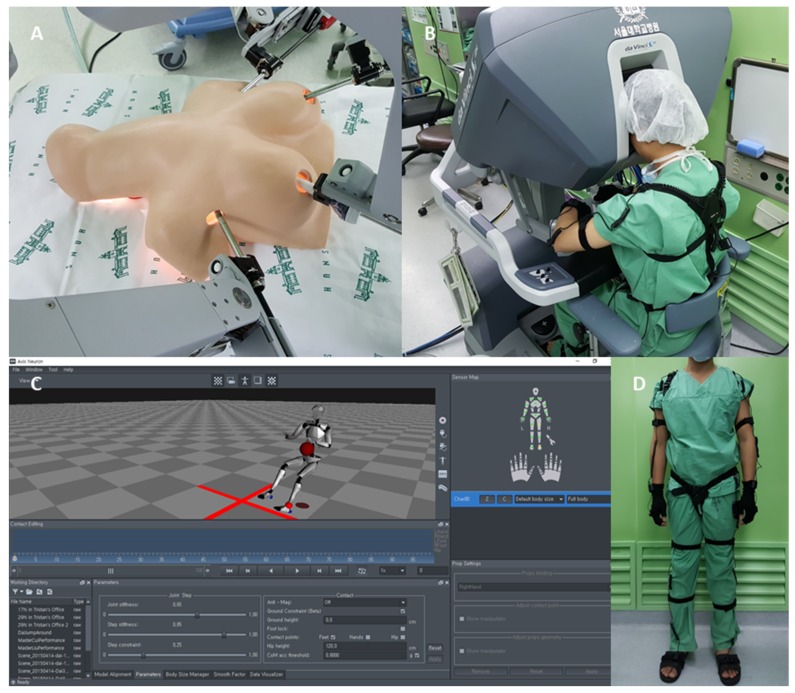
Materials used to study the usefulness of a motion capture device in surgical training. (**A**) Photograph of the BABA training model after the da Vinci robot was docked. (**B**) Photograph of a participant wearing the motion capture device and sitting at the console of the da Vinci robot. (**C**) A screenshot of the software program that was used to acquire motion data information from the Perception Neuron^®^ device. (**D**) Photograph of a participant wearing the Perception Neuron^®^ motion capture device.

The BABA training model is an anatomical model that replicates the thyroid in a 60 kg woman (Figure 2A). It consists of a top plate that replicates a woman’s chest skin and a base plate that represents the thyroid and its surrounding structures [17]. The BABA score is assessed when using the BABA training model with the da Vinci^®^ Surgical System (Intuitive Surgical, Inc., Sunnyvale, CA, USA) (Figure 2B). The BABA score is based on five surgical items, namely, lateral dissection, switching motion, identification of the recurrent laryngeal nerve, camera targeting, and contacts between instruments. The more contacts between the instruments, the lower the score is.

### 2.3. Use of the Motion Capture System to Capture the Movements of the Participants during BABA Training

Perception Neuron^®^ (Perception Neuron, Noitom, Miami, FL, USA) is a motion capture device used for many applications, including analyzing movements for video game developers, film makers, visual effects professionals, biomechanics researchers, and sports and medical analysts (https://neuronmocap.com/products/perception_neuron). Perception Neuron^®^ utilizes 9-axis sensor units called neurons, which are inertial measurement units (IMU). It measures the motion of the user using embedded data fusion, human body dynamics, and physical engine algorithms. The motion data are visualized using a software program (AXIS Neuron, Noitom, Miami, Florida, FL, USA), where the motion information can be streamed or saved (Figure 2C). Since the raw data from the software program are intended to be used for gaming purposes, they cannot be directly translated to real world coordinates. Therefore, an additional program using Unity^®^ (Unity 5.6.2f1, Unity Technologies, San Francisco, CA, USA) was developed to provide data suitable for motion analysis.

Thus, 16 neuron units of the Perception Neuron^®^ device were placed in appropriate locations on each participant so that the 3D Cartesian coordinates of both hands could be measured (Figure 2D). Each Perception Neuron^®^-wearing participant then performed the BABA training model tasks. To maximize accuracy, calibration was performed according to the manufacturer’s instructions if necessary. The motion data of the hands were recorded at 25 frames per second (fps) and were saved in a text file format for further analysis. The recorded data were analyzed by MATLAB^®^ (MATLAB R2017a, academic license, The MathWorks, Natick, MA, USA).

The recorded data included duration of recording, the 3D Cartesian coordinates of the hands, and roll, pitch, and yaw values. Five indicators that could be calculated from data from the master manipulator’s side were selected and the indicators were chosen based on the indicators from other scores. These indicators were time to complete the task (s), manipulator collision (number), movements with high acceleration (number), the economy-of-motion (mm), and used workspace (mm^3^). The time to complete the task was calculated on the basis of the first and last times recorded on the saved data. Manipulator collision was defined as occasions when the distance between the hands of the participant was below 100 mm. Movements with high acceleration were defined as occasions when the acceleration of the hands or feet of the participant exceeded 10 m/s^2^. Economy-of-motion was defined as the distance the hands or feet moved to complete the task. Used workspace was the volume covered by the movement of the participant.

### 2.4. dVSS^®^ and Standardized Tasks

dVSS^®^ is a surgical training simulation of Intuitive Surgical Inc. and is a virtual reality software program. It is an exercise tool that provides a sense of robotic surgery and helps the trainee to adapt to the robot manipulation methods before actually performing various robot operations. There are many exercises in dVSS^®^, but we evaluated the ability of the participants to perform the ring walk2 and camera targeting exercises. These two consecutive tasks were considered as one cycle. Two cycles were performed. The dVSS^®^ automatically evaluated the time taken to complete the tasks, the economy-of-motion, the number of instrument collisions, the occasions when the instrument force was excessive, the occasions when instruments were out of view, and the master workspace range. The average score of the two tasks in each cycle was used for the evaluation.

### 2.5. Calculate Participants’ Proficiency (MC Score)

We developed a MC score that effectively expresses the proficiency of the participants when performing the five standardized tasks with the BABA training model. The variables that were used in the MC score were chosen based on the similarity test result using Pearson’s correlation coefficient and their *p*-values. The five indicators of hand motion and their derivatives were compared to dVSS and BABA scores. The time to complete the task had Pearson’s correlation coefficient values of −0.789 and −0.762 (*p* < 0.001) to dVSS score and BABA score. Economy-of-motion had Pearson’s correlation coefficient value of −0.446 (*p* < 0.05) with the BABA score, used workspace/time had 0.444 (*p* < 0.05) with dVSS score, and lastly, time/used workspace had −0.583 (*p* < 0.01) with dVSS score. All the other indicators did not show statistically important correlation. Thus, the MC score was calculated as follows using four indicators with good correlation to existing scores:

MC score = (1/time × 5000) + (1/economy-of-motion × 6000) + (used workspace/time/60) − (time/used workspace × 4000)

Participants were deemed to have a high proficiency score (100 points) if: (i) they performed the standardized tasks in less than 83 seconds (this contributed 60 points to the score), (ii) they had good economy-of-motion during the five tasks, namely, the average distances between the left and right hands were less than 300 mm (this contributed 20 points to the score), and (iii) they used a large volume of the workspace per unit time (1200 mm^3^/second; this contributed 20 points to the score). Moreover, if participants had a long time per volume of used workspace (0.0025 seconds/mm^3^), they were penalized by 10 points.

### 2.6. Statistical Analysis

The five indicators derived from the hand motion data and their derivatives, such as used workspace/time, time/used workspace, economy-of-motion/time, time/economy-of-motion, were statistically analyzed against dVSS score and BABA score through the Pearson’s correlation coefficient. The similarity test result using the Pearson’s correlation coefficient and their *p*-values were used to select variables to be used in developing the MC score. Using this result, the MC score was developed, and to evaluate the correlations between the BABA and dVSS scores, between the BABA and MC scores, and between the dVSS and MC scores, Pearson’s correlation coefficient analyses was performed. To compensate for the difference in range of scores between BABA, dVSS, and MC, the scores were normalized before conducting the similarity test. Furthermore, to identify the difference between the motions that contributed to higher score, comparison between the students was performed. Based on the total score achieved by the students in BABA and dVSS in each trial, students were classified into three groups. The group two-tailed *t*-test of motion analysis indicator was performed with the high score students and low score student. All statistical analyses were conducted using SPSS (IBM SPSS Statistics for Windows, Version 21.0. IBM Corp, Armonk, NY, USA).

## 3. Results

### 3.1. Comparison of Scores for dVSS, BABA, and MC Scores

**Figure 3:**
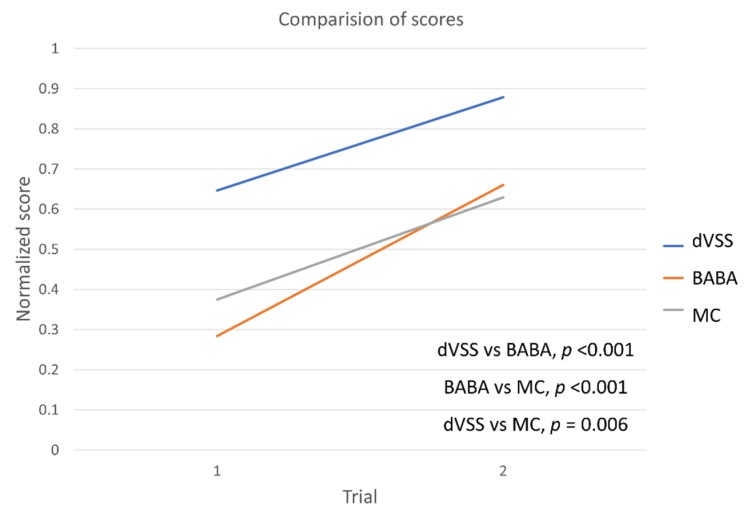
Correlations between the BABA, MC, and dVSS scores of the participants when they conducted two cycles of standardized BABA training model and dVSS tasks. Correlation analyses were performed using Pearson’s correlation coefficient.

The average with standard deviation of BABA score of the ten medical students increased from 18.1 ± 4.4 in the first cycle to 27.5 ± 5.6 in the second cycle, and the MC score increased from 44.0 ± 16.4 to 62.7 ± 21.8 (Table 1). The average dVSS score increased from 59.2 ± 22.1 in the first cycle to 80.1 ± 11.1 in the second cycle. In Figure 3, the three scores are scaled from 0 to 1 for effective visualization. The BABA and MC scores correlated significantly with each other (*p* < 0.001). The MC and dVSS scores also correlated significantly with each other (*p* = 0.006), as did the dVSS and BABA scores (*p* < 0.001).

### 3.2. Differences in Actual Body Movements between Low and High Scores

**Figure 4:**
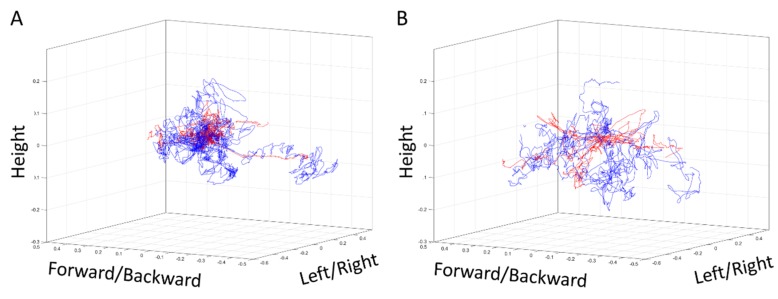
Actual trajectory of the right (blue) and left (red) hands of the participants who had (**A**) a low or (**B**) a high overall proficiency. Overall proficiency was measured by summing the dVSS, BABA, and MC scores.

The dVSS, BABA, and MC scores of the 20 dVSS + BABA trials of the ten students were summed to yield an overall score. Overall scores in the range of 0 and 130, 131 and 170, and 171 and 240 were considered to indicate low, moderate, and high overall proficiency, respectively. When these thresholds were applied to the 20 trials of the cohort, six, eight, and six were classified as having low, moderate, and high overall proficiency, respectively. In Figure 4, the actual body movements in low-scoring (Figure 4A) and high-scoring (Figure 4B) trials are shown in the 3D area. The red and blue lines show the movements of the left and right hands, respectively. The average economy-of-motion of the left hand trajectories in the low- and high-scoring groups was 369.8 and 265.4 mm, respectively; these differences were not statistically significant when analyzed by two-tailed *t*-test (*p* = 0.198). However, the economy-of-motion values of the right hand trajectories in the low- and high-scoring groups were 675.2 and 369.4 mm, respectively; this difference was statistically significant (*p* = 0.043).

## 4. Discussion

The traditional apprenticeship-based model of surgical education is increasingly being replaced by education using mannequins, cadavers, training boxes, and virtual reality simulators [13,18,19,20,21,22]. Research in this area has gradually moved from introducing simulation training to identifying the education method that most effectively teaches the requisite surgical skills. To date, however, only a few studies have sought to evaluate the effects of simulation education by analyzing the actual body movements of the trainee [23], namely, by installing a device on the trainee that converts the motions at each joint into digital data. Also, the previous study was a one that used relatively inaccurate measuring devices. In the present study on this issue, the device that was used was Perception Neuron^®^. This device was created for movement analyses by video game developers, film makers, visual effects professionals, biomechanics researchers, and sports and medical analysts. It has never been used for research in the medical field before.

BABA robotic thyroidectomy is a surgical method that removes the thyroid without leaving a scar on the neck. While it associates with a low complication rate and high surgical safety [24,25,26], it requires extensive training; about 40 cases are needed before the surgeon becomes proficient [27]. Previously, we reported that the BABA training model effectively teaches the requisite surgical skills needed for BABA robotic thyroidectomy [17]. In the present study, we used a motion capture system to assess whether body movements associated with the BABA score, which indicates the proficiency of the trainee in key BABA surgery steps.

The ten volunteer medical students were asked to undergo two cycles of dVSS and BABA model training; the motion capture device was fitted to the student during the BABA model training sessions. The proficiency with which the students conducted two dVSS and five BABA model standardized tasks in each cycle was indicated by the dVSS and BABA scores. As reported previously [17], these scores correlated significantly with each other. We then calculated the MC score, which is a composite measure of how fast the trainee completed the five BABA tasks, how economic the motions were, and how much of the workspace was used. The components of the MC score were chosen based on the correlation test result with the dVSS and BABA scores. The MC score has a maximum score of 100 indicating highest proficiency. Correlation analyses showed that both the BABA and dVSS scores correlated significantly with the MC score. To confirm these co-relationships between body movements and simulation scores, we classified the 20 trials of the students as exhibiting high, moderate, and low overall proficiency on the basis of sums of the dVSS, BABA, and MC scores for each trial. The six low-scoring and six high-scoring trials were compared in terms of economy-of-motion of the left and right hands in the 3D area. The high-scoring trials associated with significantly greater economy–of-motion in the right hand movement than the low-scoring trials (*p* = 0.043). Thus, high overall scores associated significantly with sufficient surgical movements by the dominant hand. By contrast, the low- and high-scoring trials did not differ in terms of the economy-of-motion of the left hand, which was the non-dominant hand of all participants (*p* = 0.198). These findings support the hypothesis that not only does simulation training increase proficiency scores, certain body motions associate significantly with the educational effectiveness of simulation training.

Medical education with mannequins and virtual reality simulators has three main advantages over conventional apprenticeship-based training [1]. First, training occurs in a safe environment that does not pose any risk to patients. By contrast, training by performing actual surgery creates tension in the trainee because even a small improper movement can be harmful to the patient and can cause legal problems. Second, it is possible to repeat the training, or certain aspects of the operation, as often as desired. By contrast, while most medical students are given the chance to observe the assigned task, their opportunities to perform the actual task are much more limited. Third, simulation-based training is much more focused on the individual needs of the trainee than the conventional approach, as it allows the trainee to concentrate on aspects of the operation that they are not yet good at.

These merits of simulation-based training have led to its increasing inclusion in medical curricula. Unfortunately, the current research on simulation-based training focuses strongly on the efficacy of the various training platforms. We would like to emphasize that it is important that trainees are also specifically trained to adopt the body movements that allow them to rapidly and precisely execute the operational steps. For this, it is necessary to provide the trainee with feedback about their body movements. The importance of the training method, and the motion capture device that mediates it, is demonstrated by the present study, which showed that appropriate body movements of the participants correlated with high dVSS and BABA scores.

The motion capture system that was used in the present study was introduced only recently. Our findings suggest that it will be as useful for medical education and research system as it is for exercise studies. This is supported by preliminary research showing that motion tracking devices can be used to quantify the ergonomics of medical staff during surgery [28]. Motion capture devices have also been used to evaluate the postoperative range of arm motion of patients who underwent breast surgery for breast cancer [29]. Although Perception Neuron^®^ devices are used in many fields, they have not yet been studied in medicine and medical education. Therefore, further research is needed in the future on how to use Perception Neuron^®^ in medical education. Medical education using Perception Neuron^®^ will be more effective when combined with other areas. It is possible to think about convergence with the virtual reality education which is studied recently [30,31,32,33]. Our study is the first to use Perception Neuron^®^.

This study has two limitations. First, the number of participants was small. A large-scale prospective study is needed to verify the educational effect of using the motion capture system to track the body movements of trainees. Second, we identified the dominant hand of the participants by asking them which hand was used for handwriting and eating (all stated that the right hand was dominant). In this study, it is difficult to confirm whether the movement of the right hand was statistically significant because only the right hand participated. Future studies should include the left (or non-dominant) hand as well. Furthermore, it will be necessary to identify the dominant hand by objective tests rather than by asking about the hand that was used for handwriting and eating.

## 5. Conclusions

The present study is the first to use the Perception Neuron^®^ device for body motion analysis in medical research. The study also showed that the movements of the participant during simulation-based training were as important as the training itself. At present, medical education research largely ignores the importance of teaching trainees the correct movements; instead, it largely focuses on the effectiveness of new educational methods, as reflected by exercise scores. It is likely that in the future, the educational paradigm will expand to include education on correct body movements. Devices such as Perception Neuron^®^ that analyze the 3D area of the participant will be useful for providing this feedback.

## Figures and Tables

**Table 1:** The BABA, MC, and dVSS scores of the ten participants when they conducted two cycles of standardized BABA training model and dVSS tasks.

Participant	BABA Score		MC Score		dVSS Score	
	First Cycle	Second Cycle	First Cycle	Second Cycle	First Cycle	Second Cycle
1	26	35	39.3	85.7	80	91
2	18	36	33.0	89.9	57	82
3	19	23	69.8	39.3	64	73
4	15	32	28.9	60.0	62	87
5	20	28	45.2	56.8	64	84
6	11	18	16.4	20.3	1	55
7	20	24	58.8	77.7	56	89
8	13	25	35.3	52.6	61	80
9	17	25	55.6	64.7	80	89
10	22	29	57.5	79.6	67	71
Average	18.1 ± 4.4	27.5 ± 5.6	44.0 ± 16.4	62.7 ± 21.8	59.2 ± 22.1	80.1 ± 11.1

BABA, bilateral axillo-breast approach; MC, motion capture; dVSS, da Vinci Skills Simulator.
